# Translational Health Research: where are we going?

**DOI:** 10.1590/0034-7167.2023760501

**Published:** 2023-10-06

**Authors:** Gilberto Tadeu Reis da Silva, Ludmila Anjos de Jesus

**Affiliations:** I Universidade Federal da Bahia. Salvador, Bahia, Brazil

The contributions of scientific research are undeniable for the advancement of science and for society. Therefore, more and more reflections on the impacts and applicability of studies have permeated the center of discussions at a global level, especially considering the growing increase in knowledge production. In this scenario, research translation (RT) has a notable importance insofar as the social commitment of research establishes that the knowledge arising from investigations must reverberate in fruitful and beneficial results for society, configuring a core premise of research. However, the weaknesses regarding transfer and application of knowledge from research in health services’ practice are still notorious^(1)^.

RT tunes in and correlates with the translation of knowledge, evoking, for its understanding, an imaginative variation that expresses a dynamic, interactive, multidirectional and participatory movement that involves several nuances related to health demands and characteristics of research designs, institutional actors, starting points and intersections. From this perspective, RT aims to use evidence and knowledge to close gaps and leverage transformations ethically, responsibly and effectively in health care practice.

Therefore, it is crucial to understand the tenuous distinction between knowledge translation and evidence^(2)^, given that, often, the knowledge unveiled is not transferred and concretely implemented in health practices and in health management and training policies. In this regard, the incipient understanding and implication of RT presents itself as a complex challenge, especially considering the plurality of concepts and terminologies that touch RT as well as the gaps regarding the forms of implementation^(3)^.

Therefore, RT presents itself as an area still under development and covered in complexity. This is due to the pressing need to overcome obstacles, such as the inclusion of a translational perspective in research planning, weaknesses in the researcher’s training path, resulting in unpreparedness and inexperience for RT, lack of dialogue between researchers and services, inaccessibility of evidence for services, managers and potential users of knowledge, restriction of knowledge to the academy, lack of resources and investments, in addition to the historical devaluation of qualitative research results.

On the other hand, from the identification of challenges, perspectives and initiatives to overcome them are outlined, highlighting the recovery of research’s social commitment, development of networks for dissemination and transfer of the results obtained, use of methodologies and rigorous, reproducible, and transparent research designs that include translation, reflection on the understandability of findings, increased investments, and communication strategies. Such actions favor accessibility to research and encourage a break with traditional and conservative models that tend to perpetuate unidirectional and limited patterns, which do not guarantee the realization of the public utility of the study and the strengthening of health and research systems^(1)^.

Thus, considering the aforementioned considerations as well as the spreading of knowledge disassociated from the effective incorporation of evidence in places where care is provided, which would have repercussions in advances in health care, the following reflections emerged: is there understanding, support and preparation for RT in Brazil? What paths and bridges do we need to build to ensure the effective implementation of research results? What is the future of RP? Where are we going?

The discussions raised here converge to the pressing need to (re)think research, its applications and purposes, with the intention of orchestrating them under the aegis of knowledge translation and social commitment, making RT a priority issue and intending to build paths and perspectives that value reflexivity, dissemination of knowledge and collaborative work, promoting powerful, safe and assertive transformations in health care practice for the population.

In summary, this editorial is expected to promote new reflections on the subject, contributing to re-signification of RT by reducing the distance between theory and practice, in addition to boosting engagement and concentration of efforts by researchers, research centers and services to improve transfer and use of knowledge obtained, with the aim of remodeling practices and providing subsidies to guide the decision-making process safely and reasonably, whether in clinical practice or in health policies.
